# PS-06 - IF THE ICEBERG IS MADE OF LEAD, WHY IS IT STILL FLOATING?

**DOI:** 10.1093/pubmed/fdag046.055

**Published:** 2026-07-09

**Authors:** Ms Sarah Johnson Griffiths, Dr Pallavi Patel

**Affiliations:** UK Health Security Agency; UK Health Security Agency

## Abstract

**Background:**

Despite regulation, lead remains a toxic environmental hazard, particularly in older housing. Lead is highly toxic to children, who are more at risk from the effects of lead because of their growing and developing brains, causing neurological damage, developmental delay, and hearing loss. Published data suggests that significant numbers of investigated cases of lead poisoning exhibit pica behaviour (eating non-food items), and /or learning difficulties, showing that lead poisoning disproportionately harms some of our most vulnerable population.

**Objectives:**

To explore if cases of lead toxicity reported to UKHSA for action correspond with housing risk factors and prevalence of neurodevelopmental conditions. To describe localised diagnosis of lead toxicity and explore possible reporting biases

**Methods:**

A cross-sectional descriptive study using retrospective UKHSA case and incident management data and publicly available housing related data in Northwest England from 1st January 2022 to 31st December 2023.

**Results:**

Local NorthWest reported lead toxicity cases do not wholly correspond with areas where environmental lead is expected to be higher, based on known housing risk factors, neither does it appear to reflect a geographical distribution of diagnosed neurodevelopmental conditions. Geographical distribution of North West lead cases appears to correspond with availability of specialist children services suggesting differences in clinical management and case finding.

**Conclusions:**

The study provides preliminary evidence that the number and distribution of reported cases of lead toxicity in children is not related to the well documented evidence of increased risk factors due to housing age, style or tenure. Neither does it geographically correspond to the diagnosis of neurodevelopmental conditions. Despite well documented risk factors for lead cases, we are still failing to identify cases of lead toxicity in those at greatest risk. It supports a need for wider testing and a standardisation of approaches across all paediatric services.

